# Clinical Rationale of Using Steerable Technologies for Radiofrequency Ablation Followed by Cavity Creation and Cement Augmentation in the Treatment of Painful Spinal Metastases

**DOI:** 10.3390/curroncol30040324

**Published:** 2023-04-19

**Authors:** Claudio Pusceddu, Salvatore Marsico, Daniele Derudas, Nicola Ballicu, Luca Melis, Stefano Zedda, Carlo De Felice, Alessandro Calabrese, Domiziana Santucci, Eliodoro Faiella

**Affiliations:** 1 Department of Oncological and Interventional Radiology, Businco Hospital, 09121 Cagliari, Italy; 2 Department of Radiology, Hospital del Mar, 08003 Barcelona, Spain; 3 Department of Hematology, Businco Hospital, 09121 Cagliari, Italy; 4 Nuclear Medicine Department, Businco Hospital, 09121 Cagliari, Italy; 5 Department of Radiological Sciences, Oncology and Pathology, Umberto I Hospital, Sapienza University of Rome, Viale del Policlinico 105, 00161 Rome, Italy; 6 Department of Radiology, University of Rome “Campus Bio-Medico”, Via Alvaro del Portillo, 21, 00128 Rome, Italy

**Keywords:** radiofrequency ablation: spinal metastases, quality of life, vertebral augmentation

## Abstract

(1) Background: Cement distribution after radiofrequency ablation of spinal metastases can be unpredictable due to various tumor factors, and vertebral augmentation requires advanced devices to prevent cement leakage and achieve satisfactory filling. The purpose of this study is to evaluate the safety and efficacy of a platform of steerable technologies with an articulating radiofrequency ablation (RFA) probe and targeted cavity creation before vertebral augmentation in the treatment of painful spinal metastases. (2) Methods: Sixteen patients (mean age, 67 years) underwent RFA in conjunction with vertebral augmentation after the creation of a targeted balloon cavity for metastatic spinal disease and were followed up to 6 months. Pain and functional mobility were assessed before treatment and postoperatively using the Visual Analogue Score (VAS) and Functional Mobility Scale (FMS). Complications, predictability of cement distribution, anatomical restoration, and local recurrence were collected. Technical success was defined as successful intraoperative ablation and predictable cement distribution after cavity creation without major complications. (3) Results: Sixteen patients with 21 lesions were treated for tumors involving the thoracolumbar spine. All treatments were technically successful and were followed by targeted cavity creation and vertebral augmentation. A statistically significant reduction in median VAS score was observed before treatment and 1 week after RFA treatment (*p* < 0.001). A total of six of the seven patients who reported limited painful ambulation before treatment reported normal ambulation 1 month after treatment, while the remaining patient reported no improvement. Patients who reported wheelchair use before treatment improved to normal ambulation (four/eight) or limited painful ambulation (four/eight). The improvement in mobility before and after treatment was statistically significant (*p* = 0.002). Technical success was achieved in all the combined procedures. (4) Conclusions: The combined treatment of RFA and vertebral augmentation with a steerable platform that allows the creation of a targeted cavity prior to cement injection proved to be a safe and effective procedure in our patient sample, resulting in improved quality of life as assessed by the Visual Analogue Score (VAS) and Functional Mobility Scale (FMS).

## 1. Introduction

Munk et al. described the role of percutaneous ablative therapies and cement injection in the management of metastatic spine disease, emphasizing that “It is no longer acceptable that we displace tumor into the venous system with our cement injections. We must kill the tumor first” [1].

It is widely accepted that cement augmentation alone and the exothermic reaction generated during its polymerization are not sufficient to destroy tumor cells [2], and may increase intrametastatic pressure and hypothetically push tumor cells into surrounding blood vessels [3].

The incidence of cement leakage in the treatment of spinal metastases is about 50–85% [4], and other complications include emboli due to bone marrow, tumor fragments, and bone cement. Complications of vertebroplasty are more frequent with tumor involvement rather than osteoporotic fracture (11.5% vs. 2.2–3.9%) [5]. Symptomatic leakage is frequently due to cement diffusion into the epidural space or neuroforamina.

Nowadays, radiofrequency ablation (RFA) combined with vertebral augmentation has emerged as a new treatment for pain associated with spinal metastases that can provide local tumor control and rapid pain reduction [6]. RFA performed in spinal metastases can cause coagulative necrosis of tumor cells, decrease intrametastatic pressure, and reduce complications of cement leakage for the treatment of painful, fractured, or fracture-prone neoplastic spinal lytic lesions. Recent studies have shown that the combination of RFA and vertebroplasty provides advantages over single vertebroplasty in the treatment of neoplastic vertebral lesions by improving the analgesic effect and reducing the rate of cement leakage [7].

In our past retrospective clinical trials, the benefit of the steerable radiofrequency ablation probe in combination with vertebral augmentation in the treatment of hard-to-reach bone metastases has been demonstrated to achieve local tumor control with immediate and lasting pain relief [8,9].

This study evaluates the safety and efficacy of combined percutaneous radiofrequency ablation and vertebral augmentation in the treatment of spinal metastases in a single institution, analyzing the role of the combination of ablation followed by cavity creation and cement augmentation for lesion filling. The starting hypothesis is that creating a vacuum with a high-pressure directional balloon after radiofrequency ablation could reduce intravertebral pressure, improve bone cement distribution, increase acceptable cement volume [10] and, in some cases, restore vertebral height.

The purpose of this study is to evaluate the clinical outcomes of a steerable technology platform for targeted radiofrequency ablation (STAR™ Tumor Ablation System, Merit Medical, South Jordan, UT, USA) followed by controlled, precise, and targeted cavity creation (Arcadia™ Steerable Balloon, Merit Medical) and high-viscosity cement augmentation (StabiliT^®^ MX Vertebral Augmentation System, Merit Medical) in terms of pain relief, predictable cement distribution, and absence of tumor recurrence for the treatment of patients with painful metastatic spinal tumors.

## 2. Materials and Methods

### 2.1. Study Population and Inclusion Criteria

Following institutional multidisciplinary tumor board approval, the Department of Radiation Oncology and Interventional Radiology at Businco Hospital in Cagliari, Italy, conducted a retrospective cohort study of 16 patients with spinal oligometastatic disease from May 2021 to May 2022, mainly diagnosed with breast cancer (31%, 5 patients), lung cancer (25%, 4 patients), and kidney cancer (13%, 2 patients). Eight men and eight women with an overall mean age of 67 years and an age range of 41 to 84 years were included in the study.

The starting point for patient enrollment was the presence of symptomatic vertebral metastases unresponsive to medical therapy in an oligometastatic patient with good life expectancy. Although heterogeneous, as this is the nature of the pathology, the profile of the patient amenable to treatment is widespread within the oncology population.

This retrospective study included patients meeting the following inclusion criteria: (I) pathologically confirmed spinal metastases with oligometastatic disease (number of lesions ≤ 3); (II) patients with active pain who did not respond to conventional radiation oncology therapy; (III) pathologic vertebral compression fracture (VCF) or impending fracture (bone in which a pathologic fracture appears almost certain if preventive measures are not taken [11]) evaluated as stable [12].

Exclusion criteria included patients with: (I) primary musculoskeletal tumors; (II) compression of the spinal cord and/or exiting nerve roots; (III) diffuse spinal metastatic disease (number of lesions > 3). Patients with interruptions of the posterior wall of the vertebral body were eligible for treatment.

All patients signed written informed consent according to the hospital guidelines for the radiofrequency ablation procedure combined with vertebral augmentation.

### 2.2. Radiofrequency Ablation and Targeted Void Creation with Steerable Technologies

The trajectory to reach the spinal lesion was planned, measured, and marked to establish and confirm the optimal approach and needle path under Computerized Tomography (CT) guidance. CT acquisition data were thickness 5 mm, 80–140 mA (TC system: SOMATOM Sensation, Siemens, AG, Forchheim, Germany). Procedural steps included administration of local anesthesia, planning the trajectory of the access introducer, targeted radiofrequency ablation, creation of a precise void, and vertebral augmentation.

All patients were managed with mild sedation by intravenous infusion of fentanyl citrate (0.1 mg/2 mL diluted with saline), followed by subcutaneous injection of lidocaine hydrochloride (2%) performed up to the periosteal pedicle and bony cortex.

Under CT guidance, a 10 G bevel introducer cannula was inserted through the skin into the vertebra where the lesion was located, through which an orientable PowerCURVE^®^ osteotome was inserted and used to create preferential channels for targeted placement of the STAR™ radiofrequency steerable probe.

Targeted RFA was then performed using the STAR™ RFA articulating bipolar probe, which offers the unique ability to achieve optimal positioning within the vertebra to access remote areas, including the clinically critical posterior third of the vertebral body. Embedded thermocouples along the length of the bipolar probe enabled intraoperative assessment of thermal growth to confirm and quantify the ablation zone.

**Figure 1:**
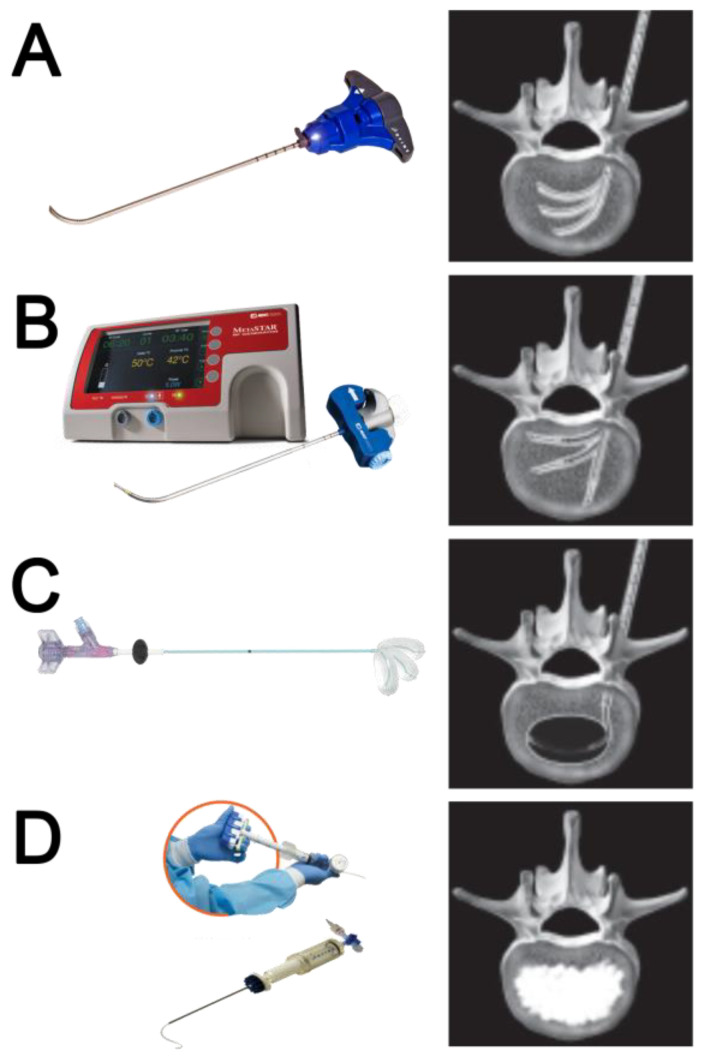
Procedural steps of RFA followed by cavity creation and cement augmentation with steerable technologies. (**A**) Channel cavity creation with PowerCURVE^®^: creation of preferential pathways into the spinal metastasis using the steerable osteotome for the following targeted placement of the RFA steerable STAR™ probe. (**B**) Targeted Radiofrequency Ablation with STAR™ probe: articulated bipolar probes allow trajectory changes within the vertebral body; thermocouples, embedded along the shaft, provide real-time feedback to quantify, adjust, and confirm the ablation zone. (**C**) Targeted cavity creation with Arcadia™: the Arcadia™ Steerable Balloon Catheter offers steerable technology to create a targeted cavity, with the intent of compacting the ablated tissue and create a void for cement delivery. (**D**) Vertebral Augmentation with StabiliT^®^ cement: high-viscosity bone cement with an extended working time of 35+ minutes, injected into the lesion with a hydraulic system providing controlled delivery.

After RFA-induced coagulative necrosis, the low-compliance, high-pressure Arcadia™ balloon was coaxially inserted into the introducer cannula, deployed into the lesion, and gently inflated with the intent to compact the ablated tissue and create a void for cement administration. The balloon was then removed, and StabiliT^®^ high-viscosity bone cement was slowly introduced into the lesion to stabilize the vertebral body. The novelty involves the creation of a cavity with a steerable device before vertebral augmentation. The steps of the procedure and the materials used are shown in Figure 1.

**Figure 2:**
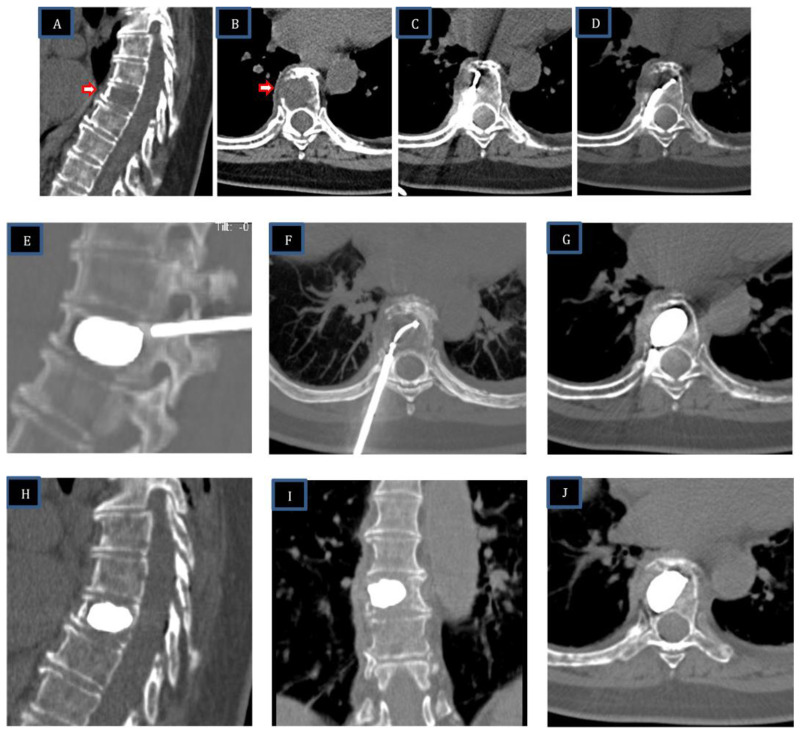
Sixty-year-old female patient with extensive metastatic lytic lesion from breast carcinoma and pathologic fracture of T8. The vertebral body (VB) of T8 has a large interruption of the right lateral wall with initial infiltration of tumor tissue outside VB. (**A**,**B**): basal CT—sagittal and axial scans show a large lytic lesion in T8 with cortical right interruption (arrow). (**C**,**D**): after channel creation with the steerable PowerCurve^®^ osteotome, two overlapping ablation zones were performed to cover the area of the lytic lesion. The first ablation zone was performed anteriorly with the STAR™ steerable RFA probe in a straight position; the second ablation zone was performed more posteriorly with the steerable RFA probe articulated across the midline. (**E**–**G**): Arcadia steerable balloon (SB) placement: deployment of the Arcadia steerable balloon along the axial-coronal plane with pedicle-to-pedicle, endplate-to-endplate inflation, creating a precise void in the ablated tissue. (**H**–**J**): Vertebral augmentation—sagittal, coronal, and axial CT scans after StabiliT^®^ high-viscosity cement with controlled hydraulic injection show no cement leakage and reconstruction of vertebral body.

**Figure 3:**
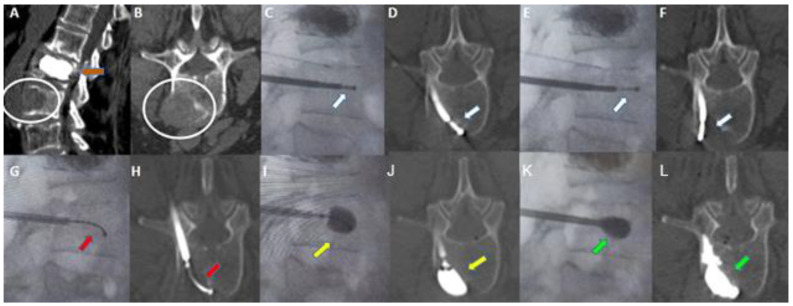
Sixty-eight-year-old woman with a history of breast cancer. (**A**,**B**): Sagittal (**A**) and axial (**B**) contrast-enhanced CT scan showing a metastatic lytic lesion of the left anterolateral portion of the L3 vertebra extending to adjacent perivertebral soft tissues (white circle in (**A**,**B**)). Previous vertebral augmentation treatment with Arcadia in L2 (orange arrow in (**A**)). (**C**,**D**): Fluoroscopy in the lateral plane (**C**) and axial CT scan (**D**). Placement of the STAR™ radiofrequency probe (white arrow) in the central portion of the lesion. (**E**,**F**): Fluoroscopy in the lateral plane (**E**) and axial CT scan (**F**). Placement of the STAR™ Radiofrequency steerable probe (white arrow) in the lateral aspect of the lesion. (**G**,**H**): Fluoroscopy in the lateral plane (**G**) and axial CT (**H**). Arcadia™ low compliance high pressure steerable balloon was inserted coaxially into the introducer cannula and deployed into the lesion (red arrow). (**I**,**J**): Fluoroscopy in the lateral plane (**I**) and axial CT (**J**). Arcadia™ low compliance high pressure steerable balloon gently inflated with a mixture of saline and iodinated contrast, with the intent of compacting the ablated tissue and creating a void for cement delivery (yellow arrow). (**K**,**L**): Fluoroscopy in the lateral plane (**K**) and axial CT (**L**). StabiliT^®^ high viscosity bone cement was slowly delivered into the lesion for the stabilization of the vertebral body (green arrow).

**Figure 4:**
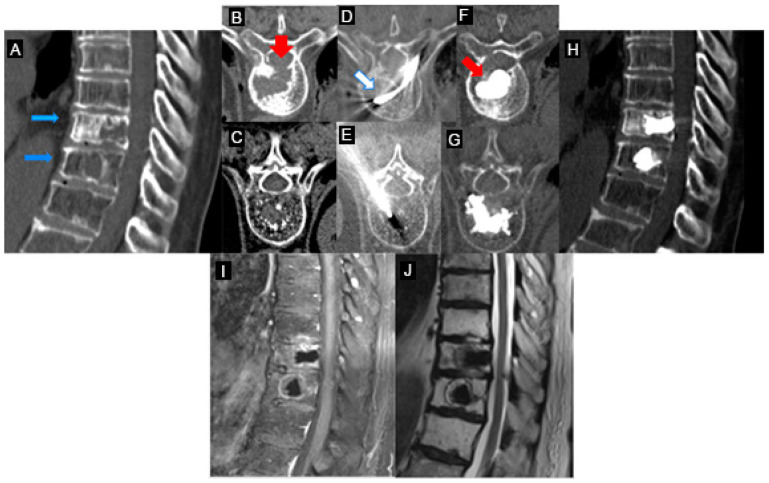
Painful pathological fractures of T8–T9 from lung carcinoma (patient #5-Table 1). (**A**–**C**): Axial and sagittal CT images of aggressive destructive osteoblastic metastasis of the T8–T9 vertebral bodies (blue arrows in (**A**)) with pathological fracture and destruction of the posterior wall of the T8 vertebra (red arrow in (**B**)). (**D**): Placement of the STAR™ radiofrequency probe (white arrow) in the central portion of the T8 vertebra lesion. (**E**,**G**): Vertebroplasty on the T9 vertebra, given the absence of posterior wall rupture. (**F**): The Arcadia™ low-compliance, high-pressure balloon was coaxially inserted into the introducer cannula and deployed into the lesion (red arrow). Note the inflation of the low-compliance Arcadia balloon after RFA and the predictable cement distribution. (**H**–**J**): Post-vertebroplasty CT (**H**) and MRI (**I**,**J**) sagittal images post procedural control T8 and T9 without complications.

Pre-procedure CT and MRI examinations were evaluated to assess the size, location, and nature (lytic/osteoblastic or mixed) of the tumor tissue, as well as the presence of any extra-vertebral extension and/or neurologic compression. Follow-up imaging (CT and/or MRI) was performed at 1, 3, and 6 months to assess local tumor control, cement distribution, targeted cavity creation, and degree of cement loss. The procedures of three patients with spinal metastasis are illustrated in Figure 2, Figure 3 and Figure 4.

### 2.3. Postoperative Follow-Up

Visual Analogue Score (VAS) data to measure pain level were collected before the procedure and 1 week, 1 month, 3 months, and 6 months after the procedure. The Functional Mobility Scale (FMS) was obtained before the procedure and 1 month after treatment to assess mobility function and walking ability. A 4-point FMS classification was used: 4, bedridden; 3, wheelchair use; 2, limited painful ambulation; 1, normal ambulation.

### 2.4. Statistical Analysis

The median and interquartile ranges (IQR) for the VAS score (before RFA and 1 week, 1 month, 3 months, and 6 months after RFA) were calculated. The evolution of the VAS score was assessed during subsequent medical visits using the Wilcoxon test for paired samples. The distribution of FMS classification before and after RFA was assessed using McNemar’s test. Analyses were performed using R v4.0.2 statistical software, and *p* values < 0.05 were considered statistically significant.

## 3. Results

**Figure 5:**
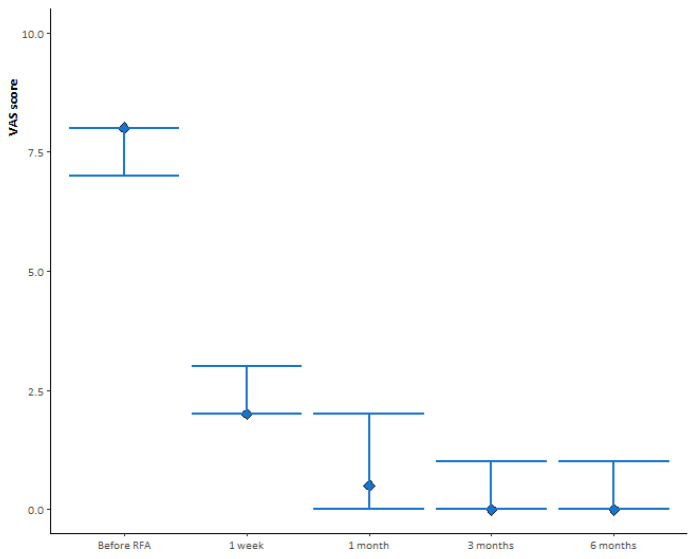
Median VAS score follow-up evaluated before and prospectively after 1 week, and 1, 3, and 6 months from the treatment.

The median (IQR) VAS score before RFA was eight (seven, eight), as shown in Figure 5. One week after treatment, the median (IQR) VAS score decreased to two (two, two), a significant change from the previous assessment (*p* < 0.001). At 1 month after treatment, we observed a further significant decrease in median (IQR) VAS score to 0.5 (0, 2) compared with 1 week after treatment (*p* = 0.002). At subsequent medical visits, the median (IQR) VAS score was zero (zero, one) at 3 months after treatment (*p* = 0.04 compared with 1 month after treatment) and zero (zero, one) at 6 months after treatment (*p* = 0.42 compared with 3 months after treatment).

**Figure 6:**
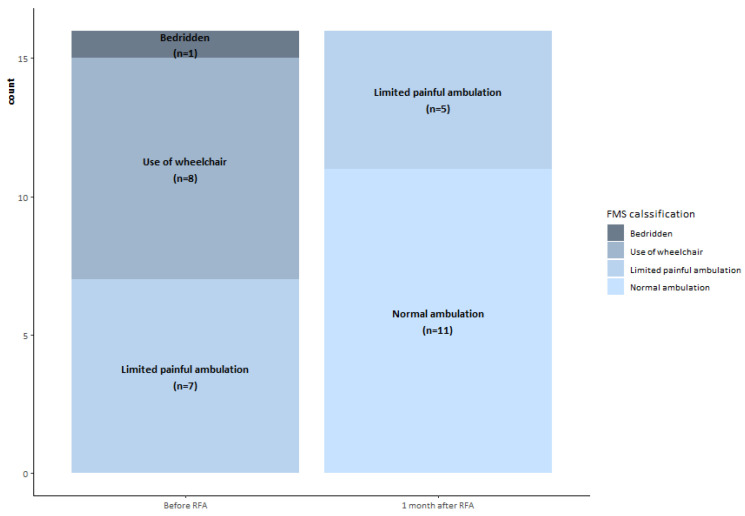
Functional Mobility Scale (FMS) classification patients rated before and 1 month after the interventional treatment.

Before treatment, one (6.2%) patient was bedridden, eight (50.0%) reported wheelchair use, seven (43.8%) reported limited painful ambulation, and none (0.0%) reported normal ambulation. One month after treatment, 68.7% of patients reported normal ambulation and 31.3% reported limited painful ambulation (Figure 6).

All but one of the patients improved their mobility as assessed by the FMS (Table 2). Six of the seven patients who reported limited painful ambulation before treatment reported normal ambulation one month after treatment, while the remaining patient reported no improvement. Patients who reported wheelchair use before treatment improved to normal ambulation (four/eight) or limited painful ambulation (four/eight). The improvement in mobility observed before and after treatment was statistically significant (*p* = 0.002).

In all combined procedures, technical success was achieved without any cement leakage. No local recurrences were observed during the 6 month follow-up. Restoration of vertebral height was achieved in 11/16 patients.

## 4. Discussion

The primary goals of RFA in combination with vertebral augmentation for metastatic spinal tumors are rapid and durable pain relief, prevention of further local tumor progression, and mechanical stability. Current treatment options include a combination of medical therapies (such as analgesics; systemic therapies including osteoclastic inhibitors such as bisphosphonates and denosumab; chemotherapy; and hormone therapy), radiation oncology (external beam radiation therapy or stereotactic body radiation therapy), and minimally invasive ablative techniques such as radiofrequency ablation.

Radiofrequency ablation is a well-established modality for the treatment of metastatic spinal disease based on predictable energy delivery and a controlled ablation zone, both properties that limit potential damage to surrounding tissues and nerves. The role of radiofrequency ablation in combination with vertebral augmentation has been included in the current guidelines from scientific societies (CIRSE Standards of Practice on Thermal Ablation of Bone Tumors [13], The American Society of Pain and Neuroscience (ASPN) Best Practices and Guidelines for the Interventional Management of Cancer-Associated Pain [14]) and guidelines in Oncology (Bone health in cancer: ESMO Clinical Practice Guidelines [15] and NCCN Adult Cancer Pain Guideline [16]). Vertebral augmentation alone has been shown to be ineffective and unsafe in the treatment of spinal metastases, as the incidence of cement leakage for spinal metastases is approximately 50% to 85% [4,17], and bone cement alone has no anti-neoplastic effect [2], increasing the risk of local recurrence [18]. Mohme et al. reported that peripheral circulating tumor cells (CTCs) are significantly increased following vertebral cement augmentation procedures, justifying the rationale for the use of a new clinical treatment option to reduce the increased release of CTCs after cement augmentation of osteolytic spinal metastases [3]. The results indicate that the addition of RFA to standard vertebral augmentation may reduce local tumor recurrence and provide additional oncological benefits compared with vertebral augmentation alone for the bone tumor patient population [18].

In our experience, technological advances have changed our clinical practice with the adoption of radiofrequency ablation for bone metastases with steerable bipolar probes and embedded thermocouples, allowing precise ablation and real-time feedback to quantify, adjust, and confirm the ablation zone intraoperatively [8,9]. Steerability in bone tumors plays a key role in overcoming suboptimal anatomy and accesses, performing adequate ablation of posterior lesions through a transpedicular approach and allowing overlapping zones through the same access. The evolution toward “precision medicine” with the steerable RF probe for bone tumors has enabled patient-specific treatment strategies using unipedicular, sequential bipedicular, or simultaneous bipedicular ablation for different clinical scenarios.

In our recent study, the role of steerable RFA devices for the treatment of hard-to-reach spinal metastases with unipedicular approaches has been highlighted [8], while Tomasian et al. reported clinical advantages in terms of local tumor control for aggressive and destructive osteolytic metastases using simultaneous steerable bipedicular RFA combined with vertebral augmentation: the goal of their treatment was to achieve confluent, coalescent, and overlapping ablation zones in the entire vertebral body (and pedicles) by achieving the target clinical volume in line with the consensus recommendations of the International Spine Radiosurgery Consortium [19].

In spinal metastatic disease, the trabecular structure is infiltrated by the presence of tumor tissue in the vertebral body, contributing to a higher intervertebral pressure. Balloon expansion and following cement injection alone may mobilize the tumoral tissue and facilitate extraosseous and epidural tumor spread [20]. In particular, simultaneous bilateral ablation with two steerable RFA probes articulated over the midline is useful for generating optimal thermodynamics given the diffuse and hyper-vascular nature of tumors, especially those of renal cell carcinoma, hepatocellular carcinoma, thyroid carcinoma, and melanoma. In metastatic spinal disease, the trabecular structure is infiltrated by the presence of tumoral tissue in the vertebral body, contributing to increased intervertebral pressure.

This study aims to evaluate the clinical rationale of precise targeted void creation and vertebral augmentation after radiofrequency ablation with a platform of steerable technologies: the rationale behind this treatment is that radiofrequency ablation contributes to coagulative necrosis of the tumor and a decrease in the intravertebral pressure of the tumor. The subsequent precise creation of balloon cavities in the dehydrated tissue after radiofrequency ablation is able to compact the ablated tissue and create a safe space for bone cement containment, promoting the predictable distribution of bone cement for optimal filling of the lesion.

In our experience, sequential procedural steps including radiofrequency ablation, creation of a targeted cavity, and injection of high-viscosity cement appear to result in lower intravertebral pressure, facilitating more consistent filing of spinal metastases and allowing more cement into the lesion, reducing the risk of leakage and improving mechanical stability. In our clinical cases, the steerable Arcadia balloon proved effective after RFA and before cement application. The creation of a cavity after ablation resulted in more predictable cement filling and increased cement volume in metastatic spinal pathologic lesions. In addition, steerability allows for adaptation to different anatomies and pathologies and is designed to inflate in the coronal plane through the vertebral body: this allows the creation of a cavity in the middle and anterior third of the vertebral body, which is biomechanically critical and promotes advantages for anatomical restoration (69%, 11 patients, achieved restoration of midline height of the vertebral body), load transfer, and stability after augmentation. Anatomical restoration is not the main goal of treatment with regard to pain relief and local tumor control, but it could be beneficial for tumor patients with long life expectancy, avoiding further adjacent fractures and/or collapse of the same pathological vertebral body.

The steerable balloon inflating in the coronal plane in the mid and anterior third of the vertebra has the advantage of restoring midline height and reducing the potential for tumor displacement to the posterior. The low-compliance Arcadia balloon resulted in less deformation during inflation, and is therefore more likely to inflate to the predetermined designed shape upon initial inflation, as well as reinflation in the same or additional vertebrae. The low compliance balloon resulted in the maintenance of the predetermined shape in sclerotic lesions as well, with the ability to optimally apply load for tissue ablated displacement with the goal of optimal cavity creation. In terms of pain relief and local tumor control, the results are in line with our previous studies [8,9]; the addition of void creation with steerable balloons appears to be a safe technique to reduce cement overflow and achieve precise cement filling.

The retrospective nature and small group of patients with heterogeneous disease and relatively short follow-up time are the main limitations of this study; however, we found a significantly better outcome than in our previous studies, a valuable approach for those metastatic bone lesions that are difficult to treat with a traditional fixed-tip needle. The study data were obtained on a small sample, as this was a pilot study of a novel technique. The improved pain control potentially achievable with the method would lead to an immediate improvement in patients’ quality of life and a reduction in the intake of pain-relieving medical therapies, with both health and economic impact [21]. Furthermore, the combined approach with the Arcadia balloon requires a thickness of the vertebral pedicle suitable for accommodating a 10-gauge coaxial needle; therefore, technically, it is necessary to select suitable candidates, unlike the standard treatment of vertebroplasty, in which smaller gauge needles can be used. It is advisable to become familiar with the new instrumentation, particularly with the modality of accessing the vertebral pedicle, of directing the needle, and of inflating the balloon, steps that require a short learning curve, which is why the technique could be more suitable for interventional radiologists skilled in spine procedures. In addition, we did not use a computational simulation approach to predict the effect of the procedure on subsequent walking conditions.

Therefore, several steps are needed to improve this study, such as enrolling more patients to level out histological differences in spine metastases and increasing the follow-up time of patients to verify the absence of local recurrences and the persistence of pain relief and mobility improvement. Models of simulation could be used to provide a reasonable estimate of the degree of successful intraoperative ablation and predictable cement distribution after cavity creation, and subsequent improvement in patient quality of life.

## 5. Conclusions

The unpredictability of cement filling and the risk of leakage still remain a crucial issue in vertebral augmentation for spinal metastases. The combined treatment of RFA and vertebroplasty with prior creation of a void can reduce intravertebral pressure, improve cement distribution in spinal metastases, and potentially reduce the occurrence of complications. The steerability feature in spinal metastatic disease results in a more robust ablative and augmentation clinical solution.

## Figures and Tables

**Table 1:** Patient and tumor characteristics and follow-up. VAS: Visual Analogue Score.

Patient	Age	Gender	Primary Cancer	Localization of Metastasis	Anatomical Restoration	VAS Pre-op	VAS 1 Week Post-op	VAS 1 Month Post-op	VAS 3 Month Post-op	VAS 6 Month Post-op	FMS Pre-op	FMS 1 Month Post-op	Status Follow-Up (Months)
1	61	M	Lung	T12, L1	yes	7	2	0	0	0	3	1	alive-12
2	60	F	Breast	T8	no	7	1	0	0	0	2	1	alive-12
3	63	F	Breast	L3	yes	8	2	0	0	0	3	1	alive-12
4	75	F	Parotid	T9, L2	yes	9	3	1	1	0	2	1	alive-11
5	73	M	Lung	T8	no	9	3	2	2	1	3	2	alive-11
6	56	M	Testicle	T12	no	8	4	3	2	2	3	2	alive-11
7	72	M	Oral cavity	T12	yes	8	3	1	1	0	2	1	alive-11
8	44	M	Pancreas	T9	yes	7	2	2	1	2	2	2	alive-11
9	76	F	Kidney	T10	no	8	2	2	1	1	3	2	alive-11
10	71	F	Breast	L1, L2	yes	9	3	0	0	0	4	1	alive-11
11	84	F	Breast	T8	yes	6	0	0	0	0	2	1	alive-10
12	72	M	Lung	T8, T9	yes	8	2	0	0	0	3	1	alive-8
13	70	F	Breast	L5	yes	7	2	2	1	1	3	2	dead-8
14	51	M	Kidney	L2	yes	8	3	0	0	0	2	1	alive-6
15	72	M	Lung	T10	no	7	1	0	0	0	3	1	alive-6
16	64	F	Ovarian	T11, T12	yes	8	3	1	0	0	2	1	alive-6

**Table 2:** Between walking performance rated on the Functional Mobility Scale (FMS) and measures of walking capacity after the treatment.

Before Treatment	After Treatment				
	Normal Ambulation	Limited Painful Ambulation	Use of Wheelchair	Bedridden	Total
Normal ambulation	0 (0.0%)	0 (0.0%)	0 (0.0%)	0 (0.0%)	0 (0.0%)
Limited painful ambulation	6 (37.5%)	1 (6.2%)	0 (0.0%)	0 (0.0%)	7 (43.8%)
Use of wheelchair	4 (25.0%)	4 (25.0%)	0 (0.0%)	0 (0.0%)	8 (50.0%)
Bedridden	1 (6.2%)	0 (0.0%)	0 (0.0%)	0 (0.0%)	1 (6.2%)
Total	11 (68.8%)	5 (31.2%)	0 (0.0%)	0 (0.0%)	16 (100.0%)

## Data Availability

The data that support the findings of this study are available on request from author, C.P. The data are not publicly available due to privacy reasons.
